# Resveratrol Upregulates Antioxidant Factors Expression and Downmodulates Interferon-Inducible Antiviral Factors in Aging

**DOI:** 10.3390/ijms26052345

**Published:** 2025-03-06

**Authors:** Iara Grigoletto Fernandes, Luana de M. Oliveira, Milena M. de Souza Andrade, Ricardo W. Alberca, Júlia Cataldo Lima, Emanuella Sarmento Alho de Sousa, Anna Julia Pietrobon, Nátalli Zanete Pereira, Anna Cláudia Calvielli Castelo Branco, Alberto José da Silva Duarte, Maria Notomi Sato

**Affiliations:** 1 Laboratory of Dermatology and Immunodeficiencies, LIM-56, Tropical Medicine Institute of Sao Paulo, Faculdade de Medicina FMUSP, Universidade de Sao Paulo, Sao Paulo 05508-000, Brazilmarisato@usp.br (M.N.S.); 2 Department of Dermatology, Laboratory of Dermatology and Immunodeficiencies, LIM-56, Faculdade de Medicina FMUSP, Universidade de Sao Paulo, Sao Paulo 05508-000, Brazil; 3 Department of Immunology, Institute of Biomedical Sciences, Universidade de Sao Paulo, Sao Paulo 05508-000, Brazil

**Keywords:** resveratrol, aging, toll-like receptors, innate immunity, inflammaging, antioxidant, antiviral

## Abstract

Immunosenescence, a process with a dysfunctional immune response that may favor infection is associated with an increase in inflammatory responses mediated by proinflammatory cytokines, characteristic of inflammaging. Aging and immunosenescence have a relationship relating to oxidative stress and inflammaging. Therefore, natural antioxidant compounds could be candidates for the control of the oxidative process. Our purpose was to evaluate the effect of resveratrol (Resv) on the antioxidant, antiviral, and anti-inflammatory responses induced by toll-like receptors (TLRs) 3, 4, and 7/8 agonists stimulation on peripheral blood mononuclear cells (PBMCs) of elderly and healthy female individuals (63–82 years old) and young and healthy female individuals (21–31 years old). Our data show that Resv may upregulate antioxidant factor expression, such as catalase (CAT) and SIRT1, in response to TLR4 and TLR7/8 agonists, similarly in both young and aged groups. Moreover, the Resv anti-inflammatory effect was detected by inhibiting IL-1β, TNF-α, and IL-10 secretion levels, as well as by the chemokines CCL2 and CCL5, induced by TLR4 and TLR7/8 stimulation. Curiously, Resv decreased antiviral genes, such as MxA, STING, and IRF7 expression, possibly by reducing the inflammatory effects of interferon-induced genes. Taken together, our results demonstrate the ability of Resv to stimulate antioxidant factors, leading to a downmodulation of the inflammatory response induced by innate immune stimulation. These findings point out Resv as a strategy to control the upregulation of inflammatory response, even in elderly individuals.

## 1. Introduction

A numerical estimate was released by the World Health Organization (WHO), pointing out that individuals aged 60 years or over in 2020 accounted for approximately 1 billion people. A forecast for the number of people aged 80 years or over should triple between 2020 and 2050 [1]. The challenges that have arisen regarding immunological changes in the elderly are associated with inflammaging, low-grade chronic systemic inflammation, and immunosenescence, leading to a progressive decline in the competence of the innate and adaptive immune systems [2]. The pandemic caused by COVID-19 has shown how the elderly can be more vulnerable to infection and how immunosenescence may become an aggravating factor [3]. Therefore, evaluating the potential of cytokine production and molecular response induced by innate response stimuli, such as activation via toll-like receptors (TLRs) in elderly people, can provide interesting data about immunosenescence/inflammaging.

TLRs are transmembrane proteins located both in the plasma membrane and in the endosomes of different cell types. These receptors can recognize different pathogens by pathogen-associated molecular patterns (PAMPs) of microorganisms or by damage-associated molecular patterns (DAMPs) and subsequently initiate an immune response [4]. TLRs are essential in the association between innate and adaptive immunity. Currently, there are ten TLRs in human cells [5].

TLR3 can recognize and respond to the stimulus of double-stranded RNA (dsRNA) viruses, whereas TLR4 is a ligand mainly for bacterial lipopolysaccharide (LPS) but also recognizes some viral structural proteins and fungal polysaccharides [6]. Activation of TLR3 leads to a unique signaling cascade, such as double-stranded RNA viruses, and these are known to induce the synthesis of type I interferons (IFN-α/β), which act as antiviral products and perform immunostimulatory activities. TLR4 initiates multiple signaling processes and expression of the NF-κB transcription factor, leading to the subsequent production of proinflammatory cytokines [4,7,8]. TLRs 7 and 8 can both be activated in response to stimuli by viruses with genetic material consisting of single-stranded RNA (ssRNA). Our group has been using it as an agonist for TLRs and as a potent modulator in different diseases [9,10,11,12,13,14,15]. Aging has shown a dysfunctional innate immune response to TLR2, TLR4, TLR3, and TLR7/8 activation agonists [16,17]. However, it is still unclear whether natural compounds in aging modulate the innate immune response triggered by TLR activation, or if this happens due to a low-grade inflammation.

Considering that aging and immunosenescence have the oxidative profile as a triggering factor, it is interesting to investigate natural compounds with antioxidant potential to modulate the oxidative process. Resv (3,5,4′-trihydroxystilbene) is a polyphenolic phytoalexin found mainly in the skin of grapes, berries, peanuts, and red wine [18,19,20,21,22]. Resv can be an effective and safe compound for the prevention and treatment of aging and age-related diseases, including cardiovascular diseases, cancer, and brain aging, basically through activation of the pathway SIRT1 activation and inflammaging modulation [23,24,25]. Recently, our group observed that Resv can attenuate neutrophil activation and decrease the release of neutrophil extracellular networks (NETs) in patients with severe symptoms of COVID-19 [26]. These data emphasize the antioxidative potential of Resv and that it can be used as an adjuvant therapy to attenuate the inflammation generated in viral infections.

Our study provides an investigation of differentially expressed genes related to oxidative stress, antiviral factors, and inflammation induced by TLR3, TLR4, and TLR7/8 agonists in PBMCs. The findings may lead to a better understanding of the control of infections in the elderly and immunosenescence.

## 2. Results

### 2.1. Effect of Resv on the Antioxidant and Antiviral Transcriptional Expression Induced by TLR Agonists Stimulation

We investigated the modulatory effect of Resv on the antiviral and antioxidant transcripts expression induced upon agonists of TLR3 (POLY(I:C)), TLR4 (LPS), and TLR7 and 8 (CL097) stimulation, representing bacterial or virus stimulation. It was assessed in PBMCs from young female (18–35 years old) and elderly (60–82 years old) healthy volunteers. We chose only females to avoid gender bias.

**Figure 1:**
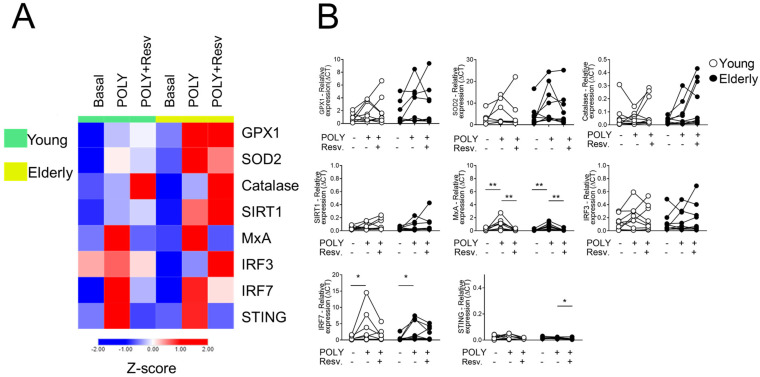
Resv downmodulates antiviral factors upon TLR3 activation. (**A**) Heatmap of antioxidant and antiviral factors expressed by PBMC stimulated with TLR agonist 3 (POLY(I:C)) and addition of Resv. The young group is colored in green, and the elderly group is colored in yellow. Red shade gene expression means above the row average, and blue shade expression means below the average. Z-score represents subtract mean, divided by standard deviation. (**B**) Comparison of the constitutive gene expression of young healthy volunteers and elderly healthy volunteers by qPCR. The relative expression of the targets was calculated in comparison to the amplification of the constitutive gene, GAPDH, and in comparison, to the non-stimulated situation. N = 9–10 individuals per group. Data are expressed as median and interquartile range. Paired Wilcoxon test: * *p* < 0.05, ** *p* < 0.001 (stimulated vs. unstimulated).

Figure 1 shows the heatmap for antioxidant (GPX1, SOD2, CAT, and SIRT1) and antiviral (MxA, IRF3, IRF7, and STING) genes upon stimulation with TLR3 agonist (POLY(I:C)). We first checked the adequate time for transcript analyses of 4 h (Supplemental Figure S1), and no interference in cell viability was perceived with Resv diluent (Supplemental Figure S2). Comparing the unstimulated situations (UNS) between the groups, both young and elderly showed a similar transcriptional profile (Figure 1A). After POLY(I:C) and Resv stimulation, an upregulation of CAT expression occurred in 31.25% of individuals. In the same situation, a downmodulation of MxA and STING was noted (Figure 1B).

**Figure 2:**
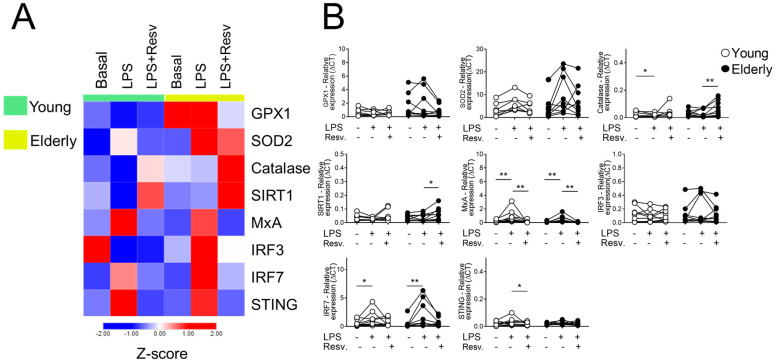
Resv upregulates CAT/SIRT1 expressions while decreasing antiviral factors upon TLR4 activation. (**A**) Heatmap of antioxidant and antiviral factors expressed by PBMCs stimulated with TLR agonist 4 (LPS) and addition of Resv. The young group is colored in green, and the elderly group is colored in yellow. Red shade gene expression means above the row average, and blue shade expression means below the average. Z-score represents subtract mean, divided by standard deviation. (**B**) Comparison of the constitutive gene expression of young healthy volunteers and elderly healthy volunteers by qPCR. The relative expression of the targets was calculated in comparison to the amplification of the constitutive gene, GAPDH, and in comparison to the non-stimulated situation. N = 9–10 individuals per group. Data are expressed as median and interquartile range. Paired Wilcoxon test: * *p* < 0.05, ** *p* < 0.001 (stimulated vs. unstimulated).

After stimulation with a TLR4 agonist (LPS), the heatmap clearly shows the antioxidant potential of Resv in the elderly group, increasing the expression of CAT in both groups and SIRT1 only in aged individuals (Figure 2A). In addition, Resv decreased MxA transcriptional levels induced by LPS in young and elderly cells and STING in the elderly group (Figure 2B).

**Figure 3:**
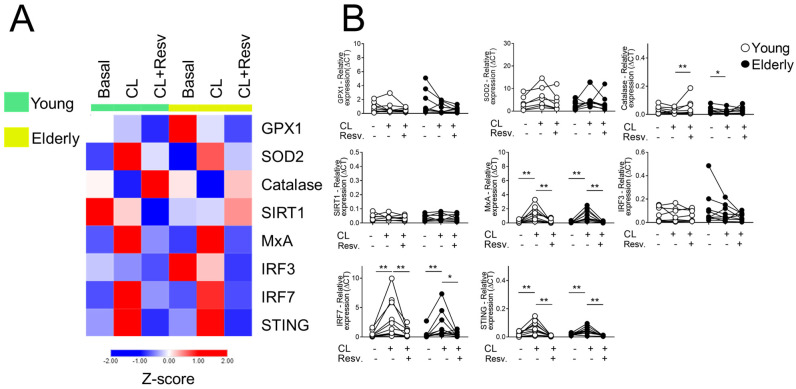
Resv upregulates CAT expressions while decreasing antiviral factors upon TLR7/8 activation. (**A**) Heatmap of antioxidant and antiviral factors expressed by PBMC stimulated with TLR agonists 7 and 8 (CL097) and addition of Resv (N = 20). The young group is colored in green, and the elderly group is colored in yellow. Red shade gene expression means above the row average, and blue shade expression means below the average. Z-score represents subtract mean, divided by standard deviation. (**B**) Comparison of the constitutive gene expression of young healthy volunteers and elderly healthy volunteers by qPCR. The relative expression of the targets was calculated in comparison to the amplification of the constitutive gene, GAPDH, and in comparison to the non-stimulated situation. N = 9–10 individuals per group. Data are expressed as median and interquartile range. Paired Wilcoxon test: * *p* < 0.05, ** *p* < 0.001 (stimulated vs. unstimulated).

Figure 3A shows that, after stimulation with CL097 in the presence of Resv, antioxidant gene expression was equated, whereas Resv decreased the expression of antiviral factors such as STING and IRF7 in both groups induced by CL097. When stimulated with CL097 and Resv, CAT showed an increased expression in young cells. Interestingly, in this same condition, the antiviral genes IRF7 and STING showed a decrease in both sample groups (Figure 3B). The comparison with the baseline values of young and old women for the mentioned targets can be seen in Supplementary Figure S3.

### 2.2. Resv Cytokines/Chemokines Induced by Innate Response Stimuli Through TLR Activation

**Figure 4:**
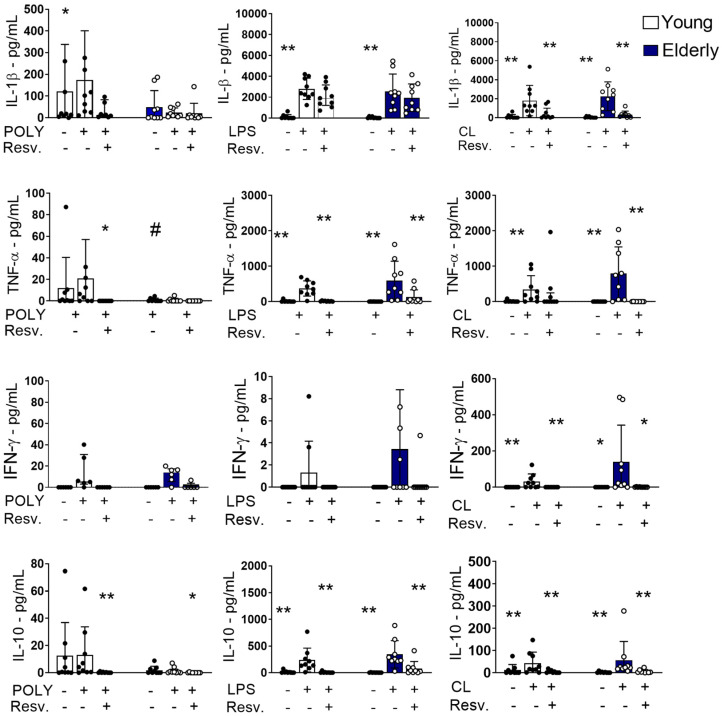
Proinflammatory cytokines induced by TLR4 and TLR7/8 stimulation were inhibited by Resv. PBMCs were incubated for 24 h with CL097 (2.5 µg/mL), LPS (1 µg/mL), POLY(I:C) (10 µg/mL), and Resv (100 µM). The production of cytokines IL-1β, TNF-α, IFN-γ, and IL-10 was assessed by flow cytometry. N = 9–10 individuals per group. Data expressed in the median and interquartile range. Unpaired Mann–Whitney test: # *p* < 0.05 (young vs. elderly). Wilcoxon paired test: * *p* < 0.05; ** *p* < 0.01 (stimulated vs. unstimulated).

Resv’s ability to modulate the function of innate response stimuli by TLR agonists was also assessed. As shown in Figure 4, Resv was able to downmodulate the production of IL-1β induced by the TLR7/8 agonist in both young and elderly groups; this effect was not verified with TLR3 or TLR4 stimulation. Dysfunctional TNF-α production was detected with TLR3 activation in the elderly group compared to the young, but not with LPS or CL097 activation. For all stimuli, Resv decreased the TNF secretion.

Again, Resv downmodulated the production of IFN-γ induced by the TLR7/8 agonist in both sample groups (Figure 4). Curiously, the anti-inflammatory cytokine IL-10 was increased in response to TLR4 and TLR7/8 agonists in the elderly group, which were downregulated by Resv, suggesting that the anti-inflammatory mechanism of action of this compound may not involve IL-10.

Monocyte chemoattractant protein-1 (MCP-1/CCL2) and CCL5/RANTES have the ability to drive the chemotaxis of myeloid and lymphoid cells. Among PBMCs, monocytes are found to be the major source of CCL2 [27] and monocytes/T-cell subsets for CCL5 [28]. As important components in the innate response and ensuing inflammatory response, we chose these chemokines for the Resv analyses.

**Figure 5:**
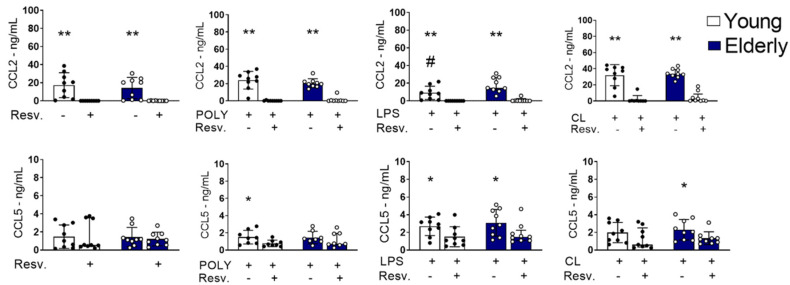
CCL2 and CCL5 induced by TLR4 and TLR3 stimulation were inhibited by Resv. PBMCs were incubated for 24 h with CL097 (2.5 µg/mL), LPS (1 µg/mL), and POLY(I:C) (10 µg/mL) in the presence of Resv (100 µM). CCL2 and CCL5 chemokines were assessed by flow cytometry. N = 9–10 individuals per group. Data expressed as median and interquartile range. One-way ANOVA test: * *p* < 0.05; ** *p* < 0.01. Unpaired Mann Whitney test: # *p* < 0.05 (stimulated vs. unstimulated).

Figure 5 shows that CCL2 was spontaneously produced, regardless of innate stimulation. Resv was able to decrease CCL2 production at unstimulated (baseline) levels, but also after TLR3, TLR4, or TRL7/8 agonist stimulation in both groups, young and old. Interestingly, cells activated with LPS from the elderly group showed a higher amount of CCL2 when compared to cells from the young group. For CCL5, Resv also induced a negative modulation after TLR4 stimulation (Figure 5).

Altogether, our data show that Resv upregulates the antioxidant factors expression, decreasing the proinflammatory cytokines TNF-α and IFN-γ, induced by LPS or CL097, including anti-inflammatory, which may inhibit antiviral factors expression. It is well known that antiviral factors are inducible by IFN-γ and trigger inflammatory reactions. Considering chemokines, Resv showed a capacity to negatively regulate the high basal levels of CCL2 in both groups, as well as the induced ones.

## 3. Discussion

Our study showed that innate immune activation induced by TLR agonists, mimicking bacterial or viral stimulation, may be controlled by in vitro Resv addition in mononuclear cells from aging/young individuals. Resv upregulates antioxidant factors, SOD2/CAT/SIRT1 axis, which contribute to suppressing the proinflammatory antiviral factors and inflammatory cytokines/chemokines induced by TLR agonists stimulation. The inflammatory process induced by interferon-induced genes (ISGs) or by the TLR signaling pathway may be protected by Resv, mainly to avoid enhancement of inflammaging status in the elderly. We chose only female volunteers to avoid bias towards male volunteers. Many studies show notable sex-based differences in immune responses to pathogens [29,30] as well as the important influence that sex hormones have on these responses [31]. Also, due to aging, their estrogen levels should be low, and none of them is taking estrogen replacement therapy. Furthermore, we chose not to work with both genders since estradiol is associated with downregulation of TLR expression, reducing oxidative stress and inhibiting proinflammatory cytokines [32].

Our data showed that even the inflammatory response triggered by LPS stimulation on PBMCs presented an upregulation of SOD2/CAT/SIRT1 expression levels, mainly in elderly individuals. This upregulation in antioxidant factors expression may represent a reflex of redox homeostasis, operating to avoid downstream signaling pathways, such as nuclear factor-κB (NF-κB), and inducing inflammatory factors in aging. More investigation is necessary to understand the mechanism involved in the elderly group to be prone to antioxidant Resv effects. One factor that we did not analyze was the gene TLR4 expression in aged mononuclear cells. Regarding the cytokines secretion induced by LPS, we found similar levels between aged and young groups. However, it has been described as evidence of both increased and decreased TLR expression and signaling in older people [16,33]. Curiously, with TLR3 agonist stimulation, only partial individuals per group were able to upregulate antioxidant factors. However, our study has the limitation of evaluating only elderly females, since the objective was to avoid heterogeneity in TLR responsiveness, considering that there are differences between the sexes concerning TLRs during aging [34]. The immune systems of males and females vary notably, especially following puberty. Specifically, women tend to be more susceptible to autoimmune conditions but have lower incidences of infections and chronic inflammatory disorders. These differences are likely influenced by sex hormones, genes located on the sex chromosomes, and gender-related behaviors [35]. Investigating whether immune responses, especially when stimulated with TLRs, function differently in elderly women compared to young women was an issue that our study intended to address, considering here the influence that sexual hormones exert on women of childbearing age and in menopausal women.

Moreover, in unstimulated PBMC conditions, Resv was able to induce CAT expression, which was enhanced with LPS stimulation. It has been described in a yellow-feathered broilers model that Resv is able to reverse the damage caused by LPS, increasing CAT and SOD activities [36]. SIRT1 induced by Resv belongs to the family of NAD-dependent histone deacetylases, involved in the regulation of the p53 gene, which is known for its protective effect and for increasing cell longevity [37,38]. Considering that ROS is derived from mitochondrial activity (90%) and immune cells (10%), perhaps the effect of resveratrol has a multi-targeted antioxidant effect, not as obvious as simply showing a decrease in ROS produced by PBMCs or cellular respiration in mitochondria. In a study that evaluated oral resveratrol supplementation in patients, a decrease in molecules highly related to oxidative stress was observed, such as malondialdehyde (MDA), which is known to be a biomarker of lipid peroxidation in plasma [39]. Other studies show that resveratrol may be involved in antioxidant action by regulating the PKA and AkT/PKB signaling pathways [40,41,42]. We observed that Resv increased SIRT-1 expression induced by LPS only in the elderly group.

Interestingly, Resv showed a dual effect, upregulating antioxidant factors, such as SOD2, CAT, and SIRT1, and decreasing antiviral factors, such as MXA and STING, regardless of the type of TLRs stimulation. There is no evidence of Resv inhibitory effects on the human antiviral factors so far; whereas, it is known that Resv has an inhibitory effect on the IFN-γ secretion, and most of the antiviral genes belong to the ISG. The inhibitory Resv effect of interferon-γ-inducible inflammatory genes may be related to a decreased STAT-1 activation [43]. Furthermore, the STING/TBK1/IRF3 axis may be critical for proinflammatory responses in rat cerebral tissue with persistent hypoperfusion, and Resv exerts its anti-inflammatory effects by suppressing STING/TBK1/IRF3 signaling [44].

Mechanistically, we consider, according to the data obtained in our study, that resveratrol is involved in inhibition of NF-κB activation by downregulation of MxA, STING, and IRF7 genes, since previous studies have shown very similar findings [45,46]. In this context, resveratrol may represent a protective agent against the harmful effects of excessive inflammation. Since IFN-γ is a potent activator of macrophages for inflammatory response and cellular response in general, overproduction of this protein may lead to hyper-responsiveness to IFN-γ, autoimmunity, and tissue damage secondary to excessive inflammation.

Considering that Resv may inhibit antiviral factors IFN-g-inducible, this compound exhibits a direct effect on RNA or DNA viruses, emphasizing Resv as a promising antiviral drug. Resv effect has been shown in anti-HSV activity [47], respiratory syncytial virus [48], zika virus [49], SARS-CoV-2 [50,51], and several other viruses. In our results, we assessed healthy aged individuals; whereas, we observed that Resv in vitro can decrease neutrophil-activated status and neutrophil extracellular traps of neutrophils in patients with severe COVID-19 [26].

The anti-inflammatory action of Resv was highlighted by the decreased production of proinflammatory cytokines, such as IL-1β, TNF-α, IFN-γ, and IL-10. Secretion levels were mainly induced by CL097, TLR7/TLR8 agonist. Double agonist likely represents an efficacious adjuvant since it is composed of TLR7-signaling pathways by IRF7 and TLR8 via NFkB. It is well known that the inhibition of NF-κB activation and NF-κB-regulated gene expression by Resv is related to the suppression of the IκB kinase activation [52]. Moreover, Resv anti-inflammatory effects may be due to SIRT-1 activation. As a deacetylase, SIRT-1 operates by blocking the TLR-4/NF-κB/STAT pathway with decreased inflammatory factor production [53].

The inhibitory effect of Resv on IL-1β and TNF-α was verified in both young and elderly individuals. However, it may show an interesting role in the control of inflammaging status during aging, especially due to the crucial role that Resv induces antioxidant effects since there is a strict relationship between oxidative stress and inflammaging. Furthermore, Resv inhibits the activation step of the NLRP3 inflammasome by suppressing mitochondrial damage and, consequently, IL-1β secretion [54].

The proinflammatory IFN-γ and anti-inflammatory IL-10 production induced by CL097 stimulation were both reduced with the addition of Resv in cell culture. These data reinforce the decreased effect on ISG, such as MxA, IRF7, and STING, all induced by Resv. IL-10 inhibition by Resv, on the other hand, was observed to be elevated by some studies [55] and inhibited by others [56], depending on the model studied.

Regarding the Resv effect on the CCL2 secretion, an inhibitory effect was already observed in unstimulated conditions. After stimulation, both CCL2/MCP-1 and CCL5 were stimulated by an agonist of TLR3, TLR4, and TLR7/8 stimuli. One of the sources of CCL2 is the monocytes in PBMCs, and CCL5s were monocytes and T-cells, again showing that Resv may control the innate/adaptive response. In fact, in Resv-treated monocytic cells, MCP-1-induced Erk phosphorylation downstream of the CCR2 receptor was dose-dependently inhibited [57].

Considering the increase in life expectancy worldwide, understanding the mechanisms involved in inflammatory and oxidative responses in the elderly becomes essential. Diseases that lead to chronic production of IL-1β and TNF-a, such as autoinflammatory syndromes and rheumatoid arthritis, can be a target for Resv, especially considering that the incidence of these conditions increases with age. In addition, considering oral resveratrol supplementation in the elderly may be indicated, since it is a potent antioxidant and anti-inflammatory agent.

## 4. Materials and Methods

### 4.1. Study Design and Casuistic

Healthy female volunteers aged between 21 and 31 years old (young group; n = 10) and healthy elderly women (63–82 years old, N = 9) were invited to participate in the study and recruited for biological sample collection. As exclusion criteria, pregnant and lactating women, patients with kidney and/or liver disease, type 1 and 2 diabetes, and positivity for HIV or hepatitis C virus were not included in the cohort. Information about the study volunteers can be found in Table 1 and Table 2. For the blood samples, collection was made in a heparin collection tube, without the need for fasting. All volunteers were informed of the research content and signed an informed-consent form, approved by the Ethics Committee on Human Research of the Faculdade de Medicina (FMUSP) of the Universidade de Sao Paulo in 2019, under CAAE number 16145719.8.0000.0065.

### 4.2. Cultures of PBMCs with TLR Agonists

After blood collection, the PBMCs were immediately separated through a density gradient using Ficoll-Paque (GE Healthcare, Uppsala, Sweden). For transcriptional analysis assays (qPCR), the cells were diluted in an RPMI medium at 1 × 10^6^ cells/well of 24-well plates cell cultures and were incubated for 4 h. This cell culture time was standardized as shown in Supplementary Figure S2. To ensure supernatants for cytokine/chemokine measurements, the cells were diluted in the RPMI medium at 2 × 10^5^ cells/well of 96-well plates cell cultures and were incubated for 24 h. They were then distributed in microplates in the RPMI culture medium with 5% AB serum (Sigma-Aldrich) and 1% penicillin/streptomycin (GibcoTM, Waltham, MA, USA) and incubated at 37 °C and 5% CO_2_, with the TLR4 agonist (receptor agonist (POLY(I:C)—10 µg/mL, InvivoGen, San Diego, CA, USA) or with the TLR 7 and 8 receptor agonist (CL097—2.5 µg/mL, InvivoGen) in the presence of Resv (100 µM—Sigma-Aldrich, St. Louis, MO, USA) or with a vehicle (acetone). For the PCR culture cell, we used 0,5% acetone (final concentration per well), and for the cytokine culture cell, we used 1% acetone (final concentration per well). In the transcript analysis assay, RNA was extracted after 4 h of culture and stored at −80 °C for a subsequent qPCR assay. For the cytokine/chemokine analysis, supernatants were collected and stored at −20 °C. The concentration of TLRs agonists, as well as the incubation time, were based on previous studies published by our group [11,15,26]. The concentration of Resv was based on a previous study showing the action of resveratrol on monocytes [57] and on a recent study published by us [26], where we showed the in vitro effect of resveratrol on the negative modulation of neutrophil extracellular traps (NET), but also, we considered that Resv displays potential effects to suppress replication of several viruses, including dengue virus, Zika virus, and influenza virus [57]. Several studies have described varying doses to exert antiviral activity, such as 50–200 μM of Resv in vitro for SARS-CoV-2 in Vero cells [50], 50 μM of RESV for respiratory syncytial virus [58], and 80 μM of RESV to induce virucidal activity against ZIKV [49]. We chose a dose of 100 μM Resv, in addition to previous laboratory data on viral infection [26], since high doses are used for antiviral effects. In our study, resveratrol concentration was defined according to a dose–response test and assay to evaluate the cytotoxicity of LDH [26].

### 4.3. Cell Viability

To evaluate the cytotoxicity of Resv and pure acetone (100%), PBMCs were incubated for 24 h with the compounds, washed twice with phosphate buffer (PBS), and incubated with the LIVE/DEAD viability marker (Invitrogen, Waltham, MA, USA), as shown in Supplementary Figure S1. The cells were incubated for 30 min, with 3 × 10^5^ cells per well, in all described situations. Cells were washed once more with PBS, and fixed in a 1% formaldehyde solution. The reading was performed in a flow cytometer (LSR Fortessa, BD, Milpitas, CA, USA). The sample acquisition was 300,000 events, and the data were analyzed using FlowJo X 10.0.6 program.

### 4.4. Real-Time PCR (qPCR)

RNeasy Plus Mini Kit (Qiagen, Hilden, Mettmann, Germany) was used following the manufacturer’s recommendations. RNA levels were measured using the NanoDrop ND-1000 spectrophotometer (ThermoScientific, Waltham, MA, USA). The amplification reaction in real-time was performed using the SYBR^®^ Green solution (Applied Biosystems, Waltham, MA, USA), reverse and forward primers for the target genes, and internal control GAPDH, designed by ThermoScientific (Table 3). Amplification was carried out using the 7500 apparatus (Applied Biosystems). The cycling protocol followed was of 10 min at 95 °C, followed by 40 cycles of 15 s at 95 °C and 60 s at 60 °C. Amplification results were visualized and analyzed using Sequence Detection. The analysis of the results was carried out using the 7500 Software v2.0.6 (Applied Biosystems), according to the delta-CT method [59].

### 4.5. Cytokine Measurement

The supernatants of PBMC cultures treated with Resv and stimuli were analyzed for the presence of TNF-α, CCL2, CCL5, IL-1β, IL-10, and IFN-γ by the CBA flow cytometry technique (Cytometric bead array BDBioscience), in a Fortessa LSR flow cytometer (BD Bioscience, LSR Fortessa, BD, Milpitas, CA, EUA). For Culture Supernatant Assay Procedure, we used Cytometric bead array (BD Bioscience, Franklin Lakes, NJ, USA). First, Cytokines Standards were reconstituted in an Assay Diluent. For the Culture Supernatant Assay Procedure, the Standards were diluted by serial dilutions, in the following order: 1:2, 1:4, 1:8, 1:16, 1:32, 1:64, 1:128, and 1:256, by using the Assay Diluent, and incubated for 15 min. Each Capture Bead suspension was vortexed before aliquoting, and 10 µL/test of each suspension was mixed. A total of 50 µL of the mixed beads was transferred to each assay tube. Standard Dilutions and test samples were added to the appropriate sample tubes (50 µL/tube). The PE Detection Reagent (50 µL/test) was then added. The samples were incubated for 3 h while protected from light. After incubation, the samples were washed with 1 mL of a Wash Buffer and centrifuged. Then, 300 µL of the Wash Buffer was added to each assay tube, and the samples were analyzed.

For the Cytometer Setup Bead Procedure, the Cytometer Setup Beads were vortexed before being added to setup tubes A, B, and C (50 µL/tube). A total of 50 µL of FITC Positive Control was added to tube B, and 50 µL of PE Positive Control was added to tube C. The tubes were incubated at room temperature for 30 min while protected from light. After incubation, 400 µL of the Wash Buffer was added to tubes B and C, and 450 µL of the Wash Buffer was added to tube A. Tubes A, B, and C were then used for cytometer setup.

The detection limit of kits for chemokines/cytokine dosage was CCL2 1.3 pg/mL, CCL5 0.002 pg/mL, TNF-α 1.2 pg/mL, IL-1b 2.3 pg/mL, IL-10 0.13 pg/mL, and IFN-g 1.8 pg/mL.

### 4.6. Statistical Analysis

The results obtained were analyzed using the GraphPad Prism 10 software (license information—serial number: GPS-2669926-e###-#####). Heatmap graphics were created using Morpheus software [60]. For unpaired data, comparisons between sample groups were performed using the non-parametric Mann–Whitney statistical test. For paired data, the Wilcoxon test was used. The level of significance considered was *p* ≤ 0.05.

## 5. Conclusions

In PBMCs, molecularly, Resv negatively regulated genes related to the antiviral response, possibly by reducing the inflammatory effects of the IFN pathway. Resv led to an increase in antioxidant genes, such as catalase and SIRT1, indicating that the anti-inflammatory signaling pathway in our study probably occurs through modulation of this pathway related to SIRT1. The production of the proinflammatory cytokines IL-1β and TNF-α and the chemokines CCL2 and CCL5 was reduced by stimulation with Resv, making it possible to conclude and demonstrate its anti-inflammatory potential.

The study limitations are related to the fact that this is an in vitro study, which may not be as faithful to what occurs in physiological situations and also to a small number of volunteers included in our trials, since recruiting elderly and healthy volunteers was difficult once the study included people without previous illnesses and of advanced age. Another relevant limitation of this study refers to the fact that most of the participants in the elderly group present overweight/obesity (IMC > 25), considering that this may lead to low-grade inflammation, and, consequently, this would possibly interfere with the results. Even so, our article provides relevant data on the dynamics involving Resv and its numerous roles in aging, as well as its anti-inflammatory, antioxidant, and even antiviral role. 

## Figures and Tables

**Table 1:** Characteristics of the young sample group.

Volunteer	Age	Medication Use	Illness	IMC
Young—1	25	No	No	24.78
Young—2	26	No	No	21.09
Young—3	27	No	No	27.24
Young—4	22	No	No	21.63
Young—5	21	No	No	29.03
Young—6	26	No	No	24.73
Young—7	26	Anticoncepcional Diclin	No	21.51
Young—8	21	No	No	24.61
Young—9	29	No	No	19.63
Young—10	30	No	No	22.23

**Table 2:** Characteristics of the elderly sample group.

Volunteer	Age	Medication Use	Illness	IMC
Elderly—1	67	Simvastatin	Controlled hypertension	29.62
Elderly—2	65	No	No	25.50
Elderly—3	73	Carvedilol	Controlled hypertension	23.24
Elderly—4	63	Rosuvastatin	Labyrinthitis	34.60
Elderly—5	64	No	No	27.48
Elderly—6	72	No	No	25.04
Elderly—7	60	No	No	29.05
Elderly—8	71	No	No	25.71
Elderly—9	63	No	No	23.56
Elderly—10	77	Ablok, corus	Controlled hypertension	33.67

**Table 3:** Primers (forward and reverse) for the target genes.

Primer	Sequence
SOD2	F: GCCCTGGAACCTCACATCAA R: TCAGGTTGTTCACGTAGGCC
GPX-1	F: TTGAGAAGTTCCTGGTGGGC R: CGATGTCAGGCTCGATGTCA
GAPDH	F: GAAGGTGAAGGTCGGAGT R: GAAGATGGTGATGGGATTTC
IRF3	F: AGAGGCTCGTGATGGTCAAGGTT R: AGAGTGGGTGGCTGTTGGAAATG
IRF7	F: TGGTCCTGGTGAAGCTGGAA R: GATGTCGTCATAGAGGCTGTTG
MxA	F: AAGCTGATCCGCCTCCACTT R: TGCAATGCACCCCTGTATACC
STING	F: ATATCTGCGGCTGATCCTGC R: GGTCTGCTGGGGCAGTTTAT
CATALASE	F: CTCCGGAACAACAGCCTTCT R: GAATGCCCGCACCTGAGTAA
SIRT1	F: TGAATATGCCAAACTTTGCTG R: GGGTGGCAACTCTGACAAAT

## Data Availability

Data are contained within the article and Supplementary Materials.

## Supplementary Materials

The following supporting information can be downloaded at https://www.mdpi.com/article/10.3390/ijms26052345/s1.

## References

[1] WHO Ageing and health World Health Organization 2022. https://www.who.int/news-room/fact-sheets/detail/ageing-and-health.

[2] Franceschi C., Bonafè M., Valensin S., Olivieri F., De Luca M., Ottaviani E., De Benedictis G. (2000). Inflamm-aging: An evolutionary perspective on immunosenescence. Ann. N. Y. Acad. Sci..

[3] Chen T., Dai Z., Mo P., Li X., Ma Z., Song S., Chen X., Luo M., Liang K., Gao S. (2020). Clinical characteristics and outcomes of older patients with coronavirus disease 2019 (COVID-19) in Wuhan, China: A single-centered, retrospective study. J. Gerontol. Ser. A.

[4] Takeda K., Kaisho T., Akira S. (2003). Toll-like receptors. Annu. Rev. Immunol..

[5] Zhang X., Zhang K., Yan L., Wang P., Zhao F., Hu S. (2023). The role of toll-like receptors in immune tolerance induced by Helicobacter pylori infection. Helicobacter..

[6] Kawai T., Akira S. (2011). Toll-like receptors and their crosstalk with other innate receptors in infection and immunity. Immunity.

[7] Vaure C., Liu Y. (2014). A comparative review of toll-like receptor 4 expression and functionality in different animal species. Front. Immunol..

[8] Takeda K., Akira S. (2015). Toll-like receptors. Curr. Protoc. Immunol..

[9] Branco A.C.C.C., Pereira N.Z., Yoshikawa F.S.Y., Oliveira L.M.D.S., Teixeira F.M.E., Oliveira L.D.M., Pietrobon A.J., Torrealba M.P., de Lima J.F., Duarte A.J.D.S. (2019). Proinflammatory profile of neonatal monocytes induced by microbial ligands is downmodulated by histamine. Sci. Rep..

[10] Pietrobon A.J., Yoshikawa F.S., Oliveira L.M., Pereira N.Z., Matozo T., de Alencar B.C., Duarte A.J., Sato M.N. (2022). Antiviral Response Induced by Toll-Like Receptor (TLR) 7/TLR8 Activation Inhibits Human Immunodeficiency Virus Type 1 Infection in Cord Blood Macrophages. J. Infect. Dis..

[11] Cardoso E.C., Pereira N.Z., Mitsunari G.E., Oliveira L.M.D.S., Ruocco R.M.S., Francisco R.P.V., Zugaib M., da Silva Duarte A.J., Sato M.N. (2013). TLR7/TLR8 activation restores defective cytokine secretion by myeloid dendritic cells but not by plasmacytoid dendritic cells in HIV-infected pregnant women and newborns. PLoS ONE.

[12] de Lollo C., de Moraes Vasconcelos D., da Silva Oliveira L.M., Domingues R., de Carvalho G.C., da Silva Duarte A.J., Zugaib M., da Silva Duarte A.J., Sato M.N. (2016). Chemokine, cytokine and type I interferon production induced by Toll-like receptor activation in common variable immune deficiency. Clin. Immunol..

[13] Manfrere K.C., Torrealba M.P., Miyashiro D.R., Oliveira L.M., de Carvalho G.C., Lima J.F., Branco A.C.C.C., Pereira N.Z., Pereira J., Sanches J.J.A. (2016). Toll-like receptor agonists partially restore the production of pro-inflammatory cytokines and type I interferon in Sézary syndrome. Oncotarget.

[14] Domingues R., de Carvalho G.C., Aoki V., da Silva Duarte A.J., Sato M.N. (2016). Activation of myeloid dendritic cells, effector cells and regulatory T cells in lichen planus. J. Transl. Med..

[15] Lima J.F., Oliveira L., Pereira N.Z., Duarte A.J., Sato M.N. (2017). Polyfunctional natural killer cells with a low activation profile in response to Toll-like receptor 3 activation in HIV-1-exposed seronegative subjects. Sci. Rep..

[16] Shaw A.C., Panda A., Joshi S.R., Qian F., Allore H.G., Montgomery R.R. (2011). Dysregulation of human Toll-like receptor function in aging. Ageing Res. Rev..

[17] Connors J., Taramangalam B., Cusimano G., Bell M.R., Matt S.M., Runner K., Gaskill P.J., DeFilippis V., Nikolich-Žugich J., Kutzler M.A. (2022). Aging alters antiviral signaling pathways resulting in functional impairment in innate immunity in response to pattern recognition receptor agonists. GeroScience.

[18] Gatouillat G., Balasse E., Joseph-Pietras D., Morjani H., Madoulet C. (2010). Resveratrol induces cell-cycle disruption and apoptosis in chemoresistant B16 melanoma. J. Cell Biochem..

[19] Lu F., Zahid M., Wang C., Saeed M., Cavalieri E.L., Rogan E.G. (2008). Resveratrol prevents estrogen-DNA adduct formation and neoplastic transformation in MCF-10F cells. Cancer Prev. Res..

[20] Zhou Z.X., Mou S.F., Chen X.Q., Gong L.L., Ge W.S. (2018). Anti-inflammatory activity of resveratrol prevents inflammation by inhibiting NF-κB in animal models of acute pharyngitis. Mol. Med. Rep..

[21] Pasinetti G.M., Wang J., Ho L., Zhao W., Dubner L. (2015). Roles of resveratrol and other grape-derived polyphenols in Alzheimer’s disease prevention and treatment. Biochim. Et Biophys. Acta (BBA)-Mol. Basis Dis..

[22] Wang D., Li S.-P., Fu J.-S., Bai L., Guo L. (2016). Resveratrol augments therapeutic efficiency of mouse bone marrow mesenchymal stem cell-based therapy in experimental autoimmune encephalomyelitis. Int. J. Dev. Neurosci..

[23] Sarubbo F., Esteban S., Miralles A., Moranta D. (2018). Effects of resveratrol and other polyphenols on Sirt1: Relevance to brain function during aging. Curr. Neuropharmacol..

[24] Zhou D.-D., Luo M., Huang S.-Y., Saimaiti A., Shang A., Gan R.-Y., Li H.-B. (2021). Effects and mechanisms of resveratrol on aging and age-related diseases. Oxidative Med. Cell Longev..

[25] Terracina S., Petrella C., Francati S., Lucarelli M., Barbato C., Minni A., Ralli M., Greco A., Tarani L., Fiore M. (2022). Antioxidant intervention to improve cognition in the aging brain: The example of hydroxytyrosol and resveratrol. Int. J. Mol. Sci..

[26] de Souza Andrade M.M., Leal V.N., Fernandes I.G., Gozzi-Silva S.C., Beserra D.R., Oliveira E.A., Teixeira F.M.E., Yendo T.M., Sousa M.d.G.T., Teodoro W.R. (2022). Resveratrol Downmodulates Neutrophil Extracellular Trap (NET) Generation by Neutrophils in Patients with Severe COVID-19. Antioxidants.

[27] Gschwandtner M., Derler R., Midwood K.S. (2019). More than just attractive: How CCL2 influences myeloid cell behavior beyond chemotaxis. Front. Immunol..

[28] Persaud A.T., Bennett S.A., Thaya L., Burnie J., Guzzo C. (2022). Human monocytes store and secrete preformed CCL5, independent of de novo protein synthesis. J. Leukoc. Biol..

[29] Forsyth K.S., Jiwrajka N., Lovell C.D., Toothacre N.E., Anguera M.C. (2024). The conneXion between sex and immune responses. Nat. Rev. Immunol..

[30] Fairweather D., Beetler D.J., McCabe E.J., Lieberman S.M. (2024). Mechanisms underlying sex differences in autoimmunity. J. Clin. Investig..

[31] Moulton V. (2018). Sex hormones in acquired immunity and autoimmune disease. Front Immunol..

[32] Khaksari M., Pourali M., Talabon S.R., Navashenaq J.G., Bashiri H., Amiresmaili S. (2024). Protective effects of 17-β-estradiol on liver injury: The role of TLR4 signaling pathway and inflammatory response. Cytokine.

[33] Boehmer E.D., Goral J., Faunce D.E., Kovacs E.J. (2004). Age-dependent decrease in Toll-like receptor 4-mediated proinflammatory cytokine production and mitogen-activated protein kinase expression. J. Leucoc. Biol..

[34] Echem C., Akamine E.H. (2021). Toll-like receptors represent an important link for sex differences in cardiovascular aging and diseases. Front. Aging.

[35] Bupp M.R.G. (2015). Sex, the aging immune system, and chronic disease. Cell Immunol..

[36] He Z., Li Y., Xiong T., Nie X., Zhang H., Zhu C. (2022). Effect of dietary resveratrol supplementation on growth performance, antioxidant capacity, intestinal immunity and gut microbiota in yellow-feathered broilers challenged with lipopolysaccharide. Front. Microbiol..

[37] Wang Z.-L., Luo X.-F., Li M.-T., Xu D., Zhou S., Chen H.-Z., Gao N., Chen Z., Zhang L.-L., Zeng X.-F. (2014). Resveratrol possesses protective effects in a pristane-induced lupus mouse model. PLoS ONE.

[38] Hodge G., Tran H.B., Reynolds P.N., Jersmann H., Hodge S. (2020). Lymphocyte senescence in COPD is associated with decreased sirtuin 1 expression in steroid resistant pro-inflammatory lymphocytes. Ther. Adv. Respir. Dis..

[39] Mahjabeen W., Khan D.A., Mirza S.A. (2022). Role of resveratrol supplementation in regulation of glucose hemostasis, inflammation and oxidative stress in patients with diabetes mellitus type 2: A randomized, placebo-controlled trial. Complement. Ther. Med..

[40] Santos M.A., Franco F.N., Caldeira C.A., de Araújo G.R., Vieira A., Chaves M.M., Lara R.C. (2021). Antioxidant effect of Resveratrol: Change in MAPK cell signaling pathway during the aging process. Arch. Gerontol. Geriatr..

[41] Franco F.N., de Cassia Cardoso L., Silva B.N.M., de Araújo G.R., Chaves M.M. (2023). Aging: Silencing the PKA and AkT/PKB signaling pathways alters the antioxidant capacity of resveratrol. Biogerontology.

[42] Yang X., Dong W.-B., Lei X.-P., Li Q.-P., Zhang L.-Y., Zhang L.-P. (2018). Resveratrol suppresses hyperoxia-induced nucleocytoplasmic shuttling of SIRT1 and ROS production in PBMC from preterm infants in vitro. J. Matern.-Fetal Neonatal Med..

[43] Chung E.Y., Kim B.H., Hong J.-T., Lee C.-K., Ahn B., Nam S.-Y., Han S.-B., Kim Y. (2011). Resveratrol down-regulates interferon-γ-inducible inflammatory genes in macrophages: Molecular mechanism via decreased STAT-1 activation. J. Nutr. Biochem..

[44] Kang N., Shi Y., Song J., Gao F., Fan M., Jin W., Gao Y., Lv P. (2022). Resveratrol reduces inflammatory response and detrimental effects in chronic cerebral hypoperfusion by down-regulating stimulator of interferon genes/TANK-binding kinase 1/interferon regulatory factor 3 signaling. Front. Aging Neurosci..

[45] Boscolo P., del Signore A., Sabbioni E., Di Gioacchino M., Di Giampaolo L., Reale M., Conti P., Paganelli R., Giaccio M. (2003). Effects of resveratrol on lymphocyte proliferation and cytokine release. Ann. Clin. Lab. Sci..

[46] Gao X., Xu Y.X., Janakiraman N., Chapman R.A., Gautam S.C. (2001). Immunomodulatory activity of resveratrol: Suppression of lymphocyte proliferation, development of cell-mediated cytotoxicity, and cytokine production. Biochem. Pharmacol..

[47] Faith S.A., Sweet T.J., Bailey E., Booth T., Docherty J.J. (2006). Resveratrol suppresses nuclear factor-κB in herpes simplex virus infected cells. Antivir. Res..

[48] Liu T., Zang N., Zhou N., Li W., Xie X., Deng Y., Ren L., Long X., Li S., Zhou L. (2014). Resveratrol inhibits the TRIF-dependent pathway by upregulating sterile alpha and armadillo motif protein, contributing to anti-inflammatory effects after respiratory syncytial virus infection. J. Virol..

[49] Mohd A., Zainal N., Tan K.-K., AbuBakar S. (2019). Resveratrol affects Zika virus replication in vitro. Sci. Rep..

[50] Yang M., Wei J., Huang T., Lei L., Shen C., Lai J., Yang M., Liu L., Yang Y., Liu G. (2020). Resveratrol inhibits the replication of severe acute respiratory syndrome coronavirus 2 (SARS-CoV-2) in cultured Vero cells. Phytother. Res..

[51] Chen X., Song X., Zhao X., Zhang Y., Wang Y., Jia R., Zou Y., Li L., Yin Z. (2022). Insights into the Anti-inflammatory and Antiviral Mechanisms of Resveratrol. Mediat. Inflamm..

[52] Holmes-McNary M., Baldwin A.S. (2000). Chemopreventive properties of trans-resveratrol are associated with inhibition of activation of the IκB kinase. Cancer Res..

[53] Wiciński M., Socha M., Walczak M., Wódkiewicz E., Malinowski B., Rewerski S., Zou Y., Li L., Yin Z. (2018). Beneficial effects of resveratrol administration—Focus on potential biochemical mechanisms in cardiovascular conditions. Nutrients.

[54] Chang Y.-P., Ka S.-M., Hsu W.-H., Chen A., Chao L.K., Lin C.-C., Hsieh C.-C., Chen M.-C., Chiu H.-W., Ho C.-L. (2015). Resveratrol inhibits NLRP3 inflammasome activation by preserving mitochondrial integrity and augmenting autophagy. J. Cell Physiol..

[55] Tong W., Chen X., Song X., Chen Y., Jia R., Zou Y., Li L., Yin L., He C., Liang X. (2020). Resveratrol inhibits LPS-induced inflammation through suppressing the signaling cascades of TLR4-NF-κB/MAPKs/IRF3. Exp. Ther. Med..

[56] Zhang Q., Huang H., Zheng F., Liu H., Qiu F., Chen Y., Liang C.L., Dai Z. (2020). Resveratrol exerts antitumor effects by downregulating CD8+ CD122+ Tregs in murine hepatocellular carcinoma. Oncoimmunology.

[57] Cicha I., Regler M., Urschel K., Goppelt-Struebe M., Daniel W.G., Garlichs C.D. (2011). Resveratrol inhibits monocytic cell chemotaxis to MCP-1 and prevents spontaneous endothelial cell migration through Rho kinase-dependent mechanism. J. Atheroscler. Thromb..

[58] Xie X.-H., Zang N., Li S.-M., Wang L.-J., Deng Y., He Y., Yang X.-Q., Liu E.-M. (2012). Resveratrol Inhibits respiratory syncytial virus-induced IL-6 production, decreases viral replication, and downregulates TRIF expression in airway epithelial cells. Inflammation.

[59] Livak K.J., Schmittgen T.D. (2001). Analysis of relative gene expression data using real-time quantitative PCR and the 2^−ΔΔCT^ method. Methods.

[60] Morpheus. https://software.broadinstitute.org/morpheus.

