# Supplementary material for: Resveratrol Upregulates Antioxidant Factors Expression and Downmodulates Interferon-Inducible Antiviral Factors in Aging
Source: Int J Mol Sci. 2025 Mar 6;26(5):2345. doi: 10.3390/ijms26052345 (PMC11900160; doi:10.3390/ijms26052345)
Supplement: Supplementary file 1 [file ijms-26-02345-s001.zip › ijms-3477466-supplementary.pdf]

Article

Resveratrol Upregulates Antioxidant Factors Expression and Downmodulates Interferon-Inducible Antiviral Factors in Aging

Iara Grigoletto Fernandes [1,2,*], Luana de M. Oliveira [1,3], Milena M. de Souza Andrade [1,2], Ricardo W. Alberca [1], Júlia Cataldo Lima [1], Emanuella Sarmento Alho de Sousa [1,3], Anna Julia Pietrobon [1,2], Nátalli Zanete Pereira [1,2], Anna Cláudia Calvielli Castelo Branco [1,3], Alberto José da Silva Duarte [1] and Maria Notomi Sato [1,2]

Supplemental Files

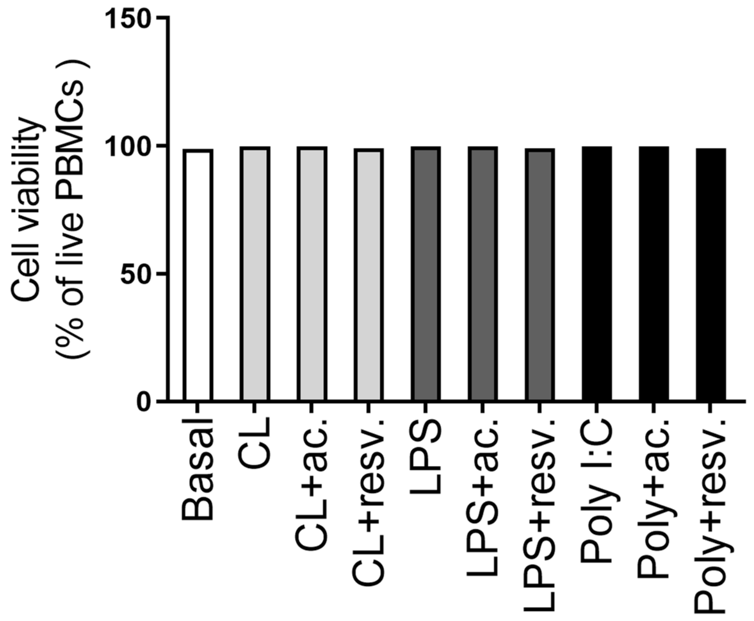

Supplementary Figure S1. Resv in cell viability. Peripheral blood mononuclear cells from 3 individuals (2 elderly and one young, n=3) were incubated for 24h with the agonists of TLR7/TLR8 (CL097, 2.5 μg/mL), TLR4 (LPS, 1 μg/mL), TLR3 (POLY(I:C), 10 μg/mL), in presence of resv (100 μM) and diluent of resv (acetone) and analyzed with LIVE/DEAD marker by flow cytometry

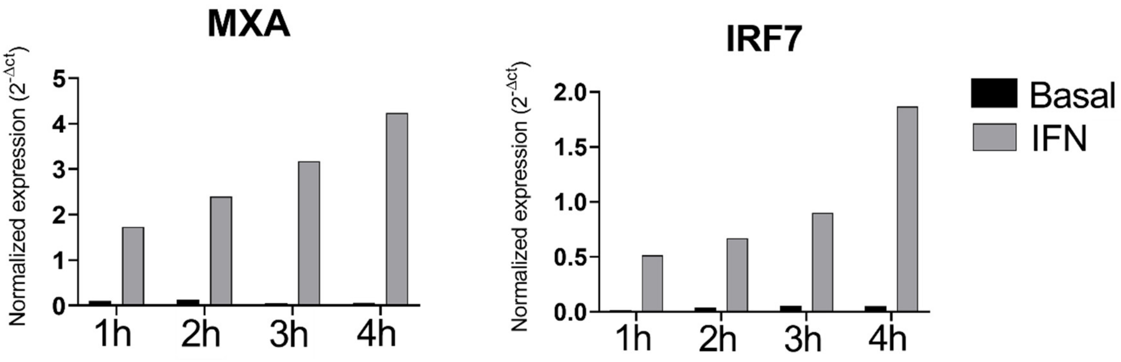

Supplementary Figure S2. kinetics for antivirals transcripts evaluation. Peripheral blood mononuclear cells from 3 individuals were incubated for 1 hour (1h), 2 hours (2h), 3 hours (3h) and 4 hours (4h), under IFN-γ stimulation. cDNA was analyzed for antiviral transcripts by q-PCR.

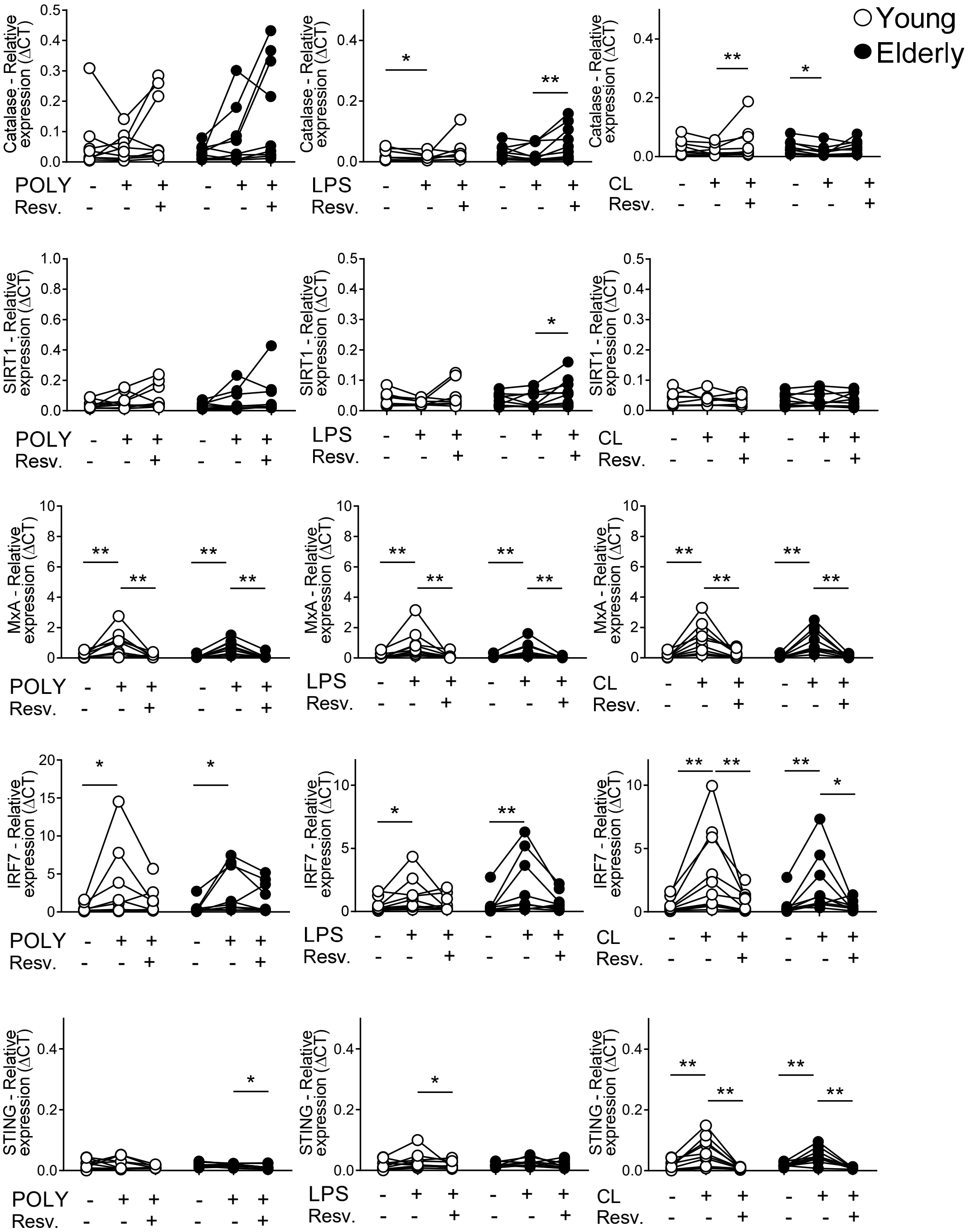

Supplementary Figure S3. Comparison of the constitutive gene expression of young healthy volunteers and elderly healthy volunteers by qPCR, including basal values. The relative expression of the targets was calculated in comparison to the amplification of the constitutive gene, GAPDH and in comparison to the non-stimulated situation. N=9-10 individuals per group. Data are expressed as median and interquartile range. Paired Wilcoxon test: *p<0.05, ** p<0.001 (Basal, Stimulated and Unstimulated).
